# Screening Analysis of Platelet miRNA Profile Revealed miR-142-3p as a Potential Biomarker in Modeling the Risk of Acute Coronary Syndrome

**DOI:** 10.3390/cells10123526

**Published:** 2021-12-14

**Authors:** Rafał Szelenberger, Michał Seweryn Karbownik, Michał Kacprzak, Karina Maciak, Michał Bijak, Marzenna Zielińska, Piotr Czarny, Tomasz Śliwiński, Joanna Saluk-Bijak

**Affiliations:** 1 Department of General Biochemistry, Faculty of Biology and Environmental Protection, University of Lodz, 90-236 Lodz, Poland; karina.maciak@edu.uni.lodz.pl (K.M.); joanna.saluk@biol.uni.lodz.pl (J.S.-B.); 2 Biohazard Prevention Centre, Faculty of Biology and Environmental Protection, University of Lodz, 90-236 Lodz, Poland; michal.bijak@biol.uni.lodz.pl; 3 Department of Pharmacology and Toxicology, Medical University of Lodz, 90-725 Lodz, Poland; michal.karbownik@umed.lodz.pl; 4 Department of Interventional Cardiology, Medical University of Lodz, 91-213 Lodz, Poland; michal.kacprzak@umed.lodz.pl (M.K.); marzenna.zielinska@umed.lodz.pl (M.Z.); 5 Department of Medical Biochemistry, Medical University of Lodz, 92-215 Lodz, Poland; piotr.czarny@umed.lodz.pl; 6 Laboratory of Medical Genetics, Faculty of Biology and Environmental Protection, University of Lodz, 90-236 Lodz, Poland; tomasz.sliwinski@biol.uni.lodz.pl

**Keywords:** blood platelets, acute coronary syndrome, microRNA, biomarker, prognostic modeling

## Abstract

Transcriptome analysis constitutes one of the major methods of elucidation of the genetic basis underlying the pathogenesis of various diseases. The post-transcriptional regulation of gene expression is mainly provided by microRNAs. Their remarkable stability in biological fluids and their high sensitivity to disease alteration indicates their potential role as biomarkers. Given the high mortality and morbidity of cardiovascular diseases, novel predictive biomarkers are sorely needed. Our study focuses for the first time on assessing potential biomarkers of acute coronary syndrome (ACS) based on the microRNA profiles of platelets. The study showed the overexpression of eight platelet microRNAs in ACS (miR-142-3p; miR-107; miR-338-3p, miR-223-3p, miR-21-5p, miR-130b-3p, miR-301a-3p, miR-221-3p) associated with platelet reactivity and functionality. Our results show that the combined model based on miR-142-3p and aspartate transaminase reached 82% sensitivity and 88% specificity in the differentiation of the studied groups. Furthermore, the analyzed miRNAs were shown to cluster into two orthogonal groups, regulated by two different biological factors. Bioinformatic analysis demonstrated that one group of microRNAs may be associated with the physiological processes of platelets, whereas the other group may be linked to platelet–vascular environment interactions. This analysis paves the way towards a better understanding of the role of platelet microRNAs in ACS pathophysiology and better modeling of the risk of ACS.

## 1. Introduction

According to the World Health Organization, cardiovascular diseases (CVDs) are the leading cause of death worldwide, representing 32% of all global deaths. Epidemiological studies have demonstrated that 85% of all deaths caused by CVDs were associated with stroke and acute coronary syndrome (ACS) [1]. ACS refers to a wide spectrum of clinical disorders that range from cardiac arrest and cardiogenic shock, with disturbed hemodynamics or electrical instability, to symptomless manifestations at the time of onset. The individual course of ACS hinders the initial diagnosis, thus inhibiting the therapeutic effect of the applied therapy [2]. In the early assessment of symptom presentation, 12-lead electrocardiography and the cardiac troponin level are still considered the gold standard in diagnosis and constitute a strong foundation in the ACS triage [2,3]. ACS biomarkers in current clinical usage are focused on the diagnosis of patients during ischemic events, thus enabling monitoring of the dynamics of myocardial ischemia [2]. However, there are no biomarkers that can be used to evaluate the potential risk of ACS development before ischemia occurs. The establishment of prognostic biomarkers would enable the implementation of preventive treatment, thus reducing the risk of ACS.

Increased interest, technological progress, and a rapidly expanding publication base have focused on novel, potential biomarkers during the last two decades, suggesting microRNAs (miRNAs) as biological molecules associated with the development of pathological conditions in various disease entities [4,5]. MiRNAs are a class of short, non-coding RNAs consisting of approximately 18–25 nucleotides. Mature miRNA sequences serve as a post-transcriptional regulator of gene expression through base-pairing with target mRNAs in the 3′-UTR region [6]. It has been suggested that about 60% of human protein-encoding genes are controlled by miRNAs, thus indicating their association with many biological processes, including proliferation, differentiation, apoptosis, and the cellular cycle [4]. The primary public repository of miRNA sequences and annotations, miRBase, currently reports that the human genome contains 2654 mature sequences and 1917 annotated hairpin precursors [7]. The screening analysis of miRNA profiles allows for the discovery of altered expression patterns, of which the identification may contribute to a better understanding of the basis of various human diseases. In 2008, Chen et al. showed that miRNAs derived from different tissues are contained in large amounts in serum with remarkable stability to harmful conditions, including a wide range of pH, repeated freeze–thaw cycles, boiling, RNAse activity, and long-term storage at room temperature. Furthermore, the presence of miRNAs was also found in blood cells [8], thus indicating the possibility of the rapid, accurate, and specific analysis of the miRNome in a non-invasive manner, emphasizing the potential role of miRNAs as biomarkers in clinical practice.

Human blood platelets play a central role in the hemostasis process, ensuring vascular stability and preventing blood loss due to vessel injury [9]. Furthermore, blood platelets constitute a major implication in thrombosis, leading to the formation of pathogenic blood clots that reduce blood flow in the vessel lumen [10]. In addition to the fact that human blood platelets are anucleate, studies have showed that they possess the transcriptome machinery necessary for maintaining biological processes [11]. It was reported that blood platelets express a vast amount of miRNAs that control their functions and reactivity [12]. Moreover, platelets contain all necessary protein enzymes delivered from megakaryocytes to convert pre-miRNA to mature sequences [13], thus constituting an excellent area for miRNA evaluation.

The main goal of our study was to perform a screening analysis of the platelet miRNome between patients diagnosed with acute coronary syndromes (ACS) and donors without any cardiovascular system disturbances via microarray technology to identify a possible alteration that could be used as a potential prognostic biomarker of ACS.

## 2. Materials and Methods

### 2.1. Chemicals

Phosphate-buffered saline (PBS) tablets were purchased from Biosigma (Venice, Italy); NaCl, NaHCO3, citric acid, sodium citrate, and NaH2PO4 were purchased from POCh (Gliwice, Poland); bovine serum albumin (BSA), Tris, NH4HCO3, glucose, KCL, and 2-[4-(2-hydroxyethyl)piperazin-1-yl]ethanesulfonic acid (HEPES) were purchased from Merck (Darmstadt, Germany).

### 2.2. Clinical Characterization of Study and Control Group

Whole blood samples were obtained from 55 patients with coronarographically confirmed ACS. However, due to the strong influence of risk factors such as impaired glycemia, high fibrinogen levels, and a highly elevated lipid profile, 5 patients were rejected from the study. Thus, 50 patients were enrolled in the study. All patients were hospitalized, diagnosed, treated, and selected for our study in the Department of Interventional Cardiology at the Medical University of Lodz, according to the current guidelines of the European Society of Cardiology. Blood samples were collected between 7:00 and 8:00 a.m. from the ulnar vein via the S-Monovette system (Sarstedt, Numbrecht, Germany) with CPDA-1 as a stabilizer. Blood was drawn from the patients immediately after the necessary examinations and treatments to ensure the patient’s safety. Patients were eligible to enroll in the study if they were under 65 years old, were not diagnosed with cancer, diabetes mellitus, or hypothyroidism, had normal kidney function, BMI < 35, a lack of connective tissue disorders, and were not addicted to narcotics and alcohol. Healthy donors were eligible for the study after medical tests which included morphology, blood levels of glucose, creatinine, aspartate transaminase (AST), alanine transaminase (ALT), total cholesterol, high-density lipoprotein (HDL), low-density lipoprotein (LDL), thyroid-stimulating hormone (TSH), and triglyceride testing. Healthy volunteers were free from any illness and were free from any medications administrated at least 2 weeks before blood collection. The study was approved by the Committee of the Ethics of Research in Human Experimentation at the University of Lodz, with resolution number 23/KBNN-UŁ/I/2017. All qualified subjects signed a written informed consent form before entering the study. All procedures performed in this study were carried out according to the Helsinki Declaration for Human Research. All clinical parameters of patients and controls are included in Table 1.

### 2.3. Blood Platelet Isolation

Blood platelets were isolated from 17 mL of fresh, whole-blood samples. Instantly after transport, tubes were centrifuged at 1200 rpm at room temperature for 15 min. To obtain the pure samples of platelets, three-quarters of platelet-rich plasma (PRP) was shifted to a fresh collection tube. Further, a magnetic separation system (Miltenyi Biotech, Bergisch Gladbach, Germany) was performed. Firstly, PRP was incubated with superparamagnetic microbeads, labeled with anti-CD45 and anti-CD235a, to deplete the leukocytes and erythrocytes, respectively. Positive separation with anti-CD61 microbeads was not recommended by the manufacturer, because of the high possibility of platelet activation. Secondly, the MS Columns responsible for the magnetic separation of labeled cells were washed 3 times with 500 µL of buffer (PBS, 0.5% BSA, 2 mM Citrate) and PRP was applied into the MS Column. Unlabeled, cleaned PRP, which passed through to the new, fresh tube, was then centrifuged at 1400 rpm at room temperature for 15 min to obtain a blood platelet pellet. Isolated blood platelets were washed three times with modified Tyrode’s buffer (127 mM NaCl, 2.7 mM KCl, 0.5 mM NaH_2_PO_4_, 12 mM NaHCO_3_, 5 mM HEPES, 5.6 mM glucose, pH 7.4) to remove potential contamination by plasma. In the final step, each platelet pellet was suspended in RNAlater (Thermo Fisher Scientific, Waltham, MA, USA) to preserve the integrity of the RNA. After the collection, samples were stored at −80 °C for further analysis.

### 2.4. RNA Isolation and Synthesis of Complementary DNA (cDNA)

The extraction of RNA from previously collected and purified platelet samples was performed using a commercially available Isolate II RNA Mini Kit (Bioline, London, UK). Because of the density of the RNAlater Solution (Thermo Fisher Scientific, Waltham, MA, USA) used during platelet isolation, an equal volume of ice-cold PBS was added and centrifuged at 5000 rpm for 5 min in 4 °C immediately before the RNA extraction to remove the RNAlater. All steps were performed according to the manufacturer’s protocols. After RNA isolation, RNA integrity was examined in TapeStation 2200 using High-Sensitivity RNA ScreenTape (Agilent, Santa Clara, CA, USA). Study and control samples achieved ~8 points on the 1–10 RINe scale. Subsequently, eluted RNA was reverse-transcribed to cDNA using a Taqman™ Advanced miRNA cDNA Synthesis Kit (Thermo Fisher Scientific, Waltham, MA, USA) with cel-miR-39-3p (Thermo Fisher Scientific, Waltham, MA, USA) as an exogenous control. The final concentration of exogenous control in each sample was 10 pM. Undiluted, transcribed cDNA was stored at −80 °C before real-time quantitative PCR (RT-qPCR) analysis.

### 2.5. Microarray Analysis

Microarray analysis was performed using an Agilent miRNA Microarray SurePrint G3 Human miRNA r21 Array Kit (Agilent, Santa Clara, CA, USA), which ensures a high-throughput system with optimal sequence discrimination and assessment of sensitivity and specificity. The applied microarray slide contained 2549 human miRNAs sourced in the miRBase Database Release 21.0. Unique SurePrint inkjet technology provided a synthesis of 40 to 60-mer oligonucleotides anchored directly on the slide with an array. The microarray contained approximately 15,000 printed probes, randomly placed to ensure the availability of all miRNAs to the tested sample. To perform an experiment, total RNA samples stored at −80 °C were transferred in dry ice to the Institute of Biology in Jan Kochanowski University in Kielce, Poland. The protocol consisted of several steps, including preparing spike-in solutions for the generation of fluorescently labeled miRNAs; and sample dephosphorylation, denaturation, ligation, and drying were carried out. After all steps were performed, microarray slides were put into the SureHyb chamber cover and inserted into the oven’s rotating rack. Samples were hybridized at 55 °C for 24 h and 20 rpm. Microarray slides were then washed and scanned. To avoid possible complications after washing, slides were immediately put into the ozone-barrier slide cover. Slides were scanned in Agilent G2565C using Feature Extraction software (FE Version 10.10.1.1) with grid number 070156_D_F_20141006. Subsequently, detailed bioinformatic analysis was performed in GeneSpring GX software (Agilent, Santa Clara, CA, USA). All stages were performed according to the manufacturer’s protocol.

### 2.6. Validation of Selected miRNAs with Real-Time PCR

RT-qPCR analysis was performed for the selected miRNAs: hsa-miR-223-3p (Assay ID: 477983_mir), hsa-miR-142-3p (477910_mir), hsa_miR-126-3p (477887_mir), hsa-miR-21-5p (477975_mir), hsa-miR-107 (478254_mir), hsa-miR-28-5p (478000_mir), hsa-miR-221-3p (477981_mir), hsa-miR-98-5p (478590_mir), hsa-let-7f-5p (478578_mir), hsa-let-7d-5p 478439_mir), hsa-let-7g-5p (478580_mir), hsa-miR-146a-5p (478399_mir), hsa-miR-301a-3p (477815_mir), hsa-miR-130b-3p (477840_mir), hsa-miR-338-3p (478037_mir), hsa-miR-191-5p (477952_mir), cel-miR-39-3p (Sequence 5′ to 3′: (RNA)-Phos-UCACCGGGUGUAAAUCAGCUUG). Taqman™ Advanced miRNA Assays (Thermo Fisher Scientific, Waltham, Massachusetts, USA), Taqman™ Fast Advanced Master Mix (Thermo Fisher Scientific, Waltham, MA, USA), Molecular-Grade Water (Thermo Fisher Scientific, Waltham, MA, USA), and 10-times diluted cDNA of the studied samples were used to quantify each miRNA molecule. To evaluate the quality of cDNA synthesis, the Ct value for an exogenous control was determined and compared in all analyzed samples. For normalization of the expression level, the mean value of endogenous (hsa-miR-191-5p) and exogenous (cel-miR-39-3p) controls was calculated and used as a reference for the ΔCt calculation. RT-qPCR analysis was performed using a CFX96TM Real-time PCR Detection System Thermal Cycler (Bio Rad Laboratories Inc., Hercules, CA, USA). The conditions set for the analysis consisted of polymerase activation (20 s, 95 °C) followed by denaturation (3 s, 95 °C), and extension (30 s, 60 °C) in 40 cycles. The expression of miRNAs was presented as −ΔCt. The difference in miRNA expression between ACS patients and healthy controls was tested with Student’s *t*-test. The fold-change of miRNA expression, together with its 95% confidence intervals (95%CI), was based on the 2^−ΔΔCt^ method.

## 3. Results

### 3.1. Screening of Platelet miRNome with Microarrays

**Figure 1:**
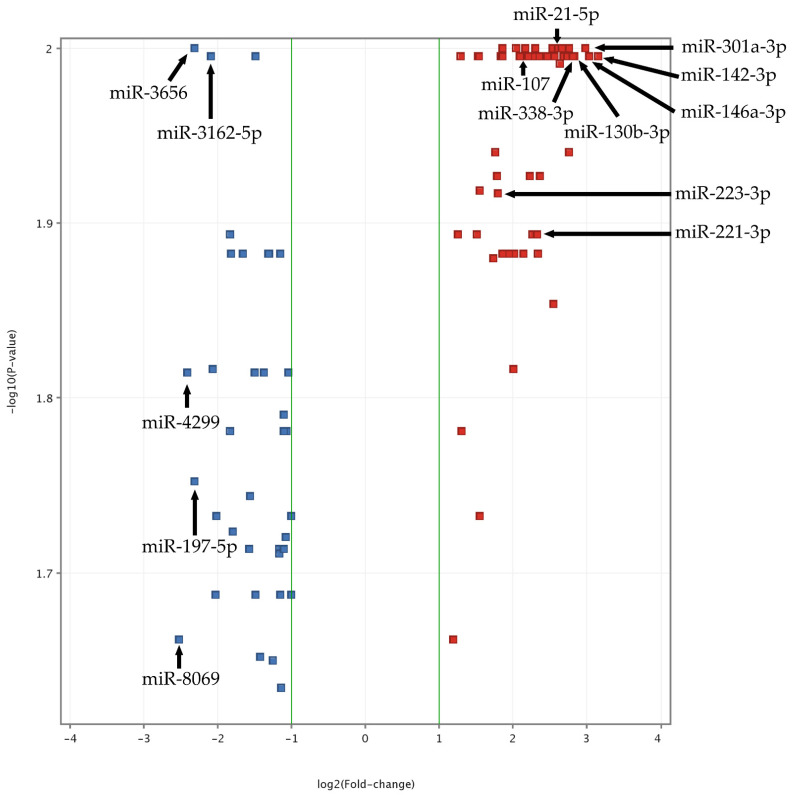
Volcano plot of ACS vs. Control miRNA microarray analysis. The data for all miRNAs are plotted as log_2_(Fold-change) vs. the −log_10_(*p*-value). A fold-change threshold is presented as green lines. The 5 most upregulated (miR-301a-3p, miR-142-3p, miR-146a-3p, miR-130b-3p, miR-338-3p) and downregulated (miR-8069, miR-4299, miR-3656, miR-197-5p, miR-3162-5p) miRNAs, selected based on fold-change values, are indicated by black arrows. Furthermore, statistically significant miRNAs, validated by qRT-PCR, are also presented (miR-142-3p, miR-107, miR-338-3p, miR-223-3p, miR-21-5p, miR-130b-3p, miR-301a-3p, and miR-221-3p). The altered expressed miRNAs between studied groups with the highest significance (the lowest *p*-value) are located at the top of the plot. MiRNAs demonstrating large fold-change values are plotted outside of the vertical threshold lines. A greater distance from the center indicates a greater fold-change. Red square points on the plot represent upregulated miRNAs, whereas blue square points on plots represent downregulated miRNAs.

To obtain the most reproducible and statistically significant findings and to exclude false-positive or false-negative results, quality control analysis was performed in study and control groups. Data were normalized using a percentile shift algorithm, without baseline transformation. Statistical analysis was performed using the moderated *t*-test with Benjamini–Hochberg FDR correction with a *p*-value cut-off of 0.05. The obtained results showed that 107 miRNA molecules had significantly altered expression in ACS patients compared to the control group. In the next step, data were filtered by fold-change value with cut-off of 2. After filtration, we found that 91 miRNAs had significantly different expression between the groups (Supplementary Figure S1, “The heatmap of miRNAs expression in microarray analysis”). Obtained results are presented in a volcano plot (Figure 1). To assess the biological functions (BFs) of the analyzed miRNAs, six databases responsible for finding target genes were used (miRDB, microRNAorg, PITA, PICTAR, TARBASE, and TARGETSCAN). To provide the most reproducible results of the predicted targets, we considered only entities supported by all of the abovementioned target prediction databases. All genes associated with particular miRNAs were studied in DAVID Bioinformatics Resources 6.8 [14]. miRNAs and genes were selected based on the OMIM Database [15], KEGG Pathways [16], Gene Ontology [14], and available publications. For validation of the obtained results by RT-qPCR, we selected 15 miRNAs presenting altered expression in ACS patients compared to healthy volunteers and demonstrating an association with platelet function.

### 3.2. Validation of Blood Platelet miRNA by RT-qPCR

**Figure 2:**
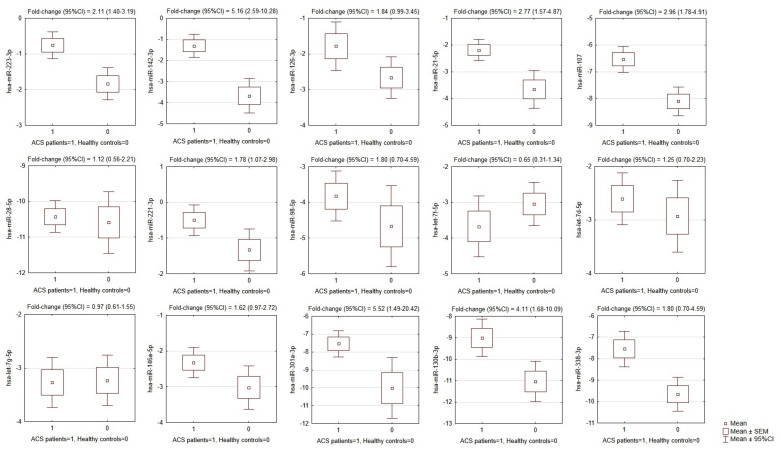
Comparison of miRNA expression in platelets of ACS patients and matched healthy controls. OY axes depict −ΔCt values for each given miRNA, presented graphically as the mean, SEM, and 95% CI of expression estimate. Above individual box-plots, the fold-change (95% CI) of miRNA expression in ACS patients in relation to its expression in healthy controls is presented. ACS—acute coronary syndrome, SEM—standard error of mean, CI—confidence interval.

The results showed that of the 15 selected miRNAs from the microarray analysis, the expression of eight miRNAs was significantly augmented in ACS patients compared to healthy controls. Overexpression was found for hsa-miR-142-3p (*p* < 0.0001); hsa-miR-107 (*p* <0.0001); hsa-miR-338-3p (*p* = 0.0004), hsa-miR-223-3p (*p* = 0.0004), hsa-miR-21-5p (*p* = 0.0005), hsa-miR-130b-3p (*p* = 0.0023), hsa-miR-301a-3p (*p* = 0.011), and hsa-miR-221-3p (*p* = 0.027). The mean values of −ΔCt, SEMs, and fold-changes are presented in Figure 2.

### 3.3. Statistical Analysis and Modeling of Potential Biomarkers

Biochemical parameters were transformed via the Box–Cox procedure to maintain the normal distribution of the parameters. Receiver operating characteristic (ROC) curves were used to illustrate model performance to differentiate ACS patients from healthy controls and areas under the ROC curves (AUC) with 95%CI values were estimated. Cut-off points were proposed based on the maximization of Youden’s index. The colinearity of miRNAs was tested with the use of exploratory factor analysis (EFA) and Pearson’s *r* correlation matrix analysis. The number of factors in EFA was established based on the scree-plot analysis and the eigenvalue-more-than-one criterion. The factor differentiation in EFA was achieved by means of raw varimax factor rotation. Binary logistic regression was used to build a multivariate model to differentiate ACS patients from healthy controls. Predictors were selected based on the step-wise procedure and with the help of Pearson’s *r* correlation matrix analysis. The models were evaluated with the use of Akaike and Bayesian information criteria, as well as Cox–Snell and Nagelkerke’s pseudo-R^2^. To assess the goodness-of-fit, the Hosmer–Lemeshow chi-squared test was performed. Ten-fold cross validation was used as an internal model validation technique. *p*-values below 0.05 were considered statistically significant. The analysis was performed using STATISTICA 13.3 Software (StatSoft; Tulsa, OK, USA).

**Figure 3:**
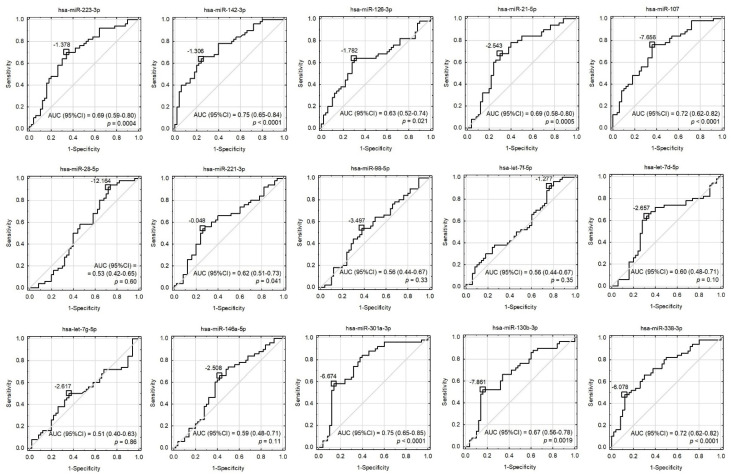
Receiver operating characteristic (ROC) curves for univariate models involving all the tested miRNAs. Cut-off points were proposed based on the maximization of Youden’s index and their values were presented as −ΔCt. The area under the ROC curves (AUC), their 95% confidence intervals (95%CI), and the tests for AUC differences from 0.5 were displayed.

**Figure 4:**
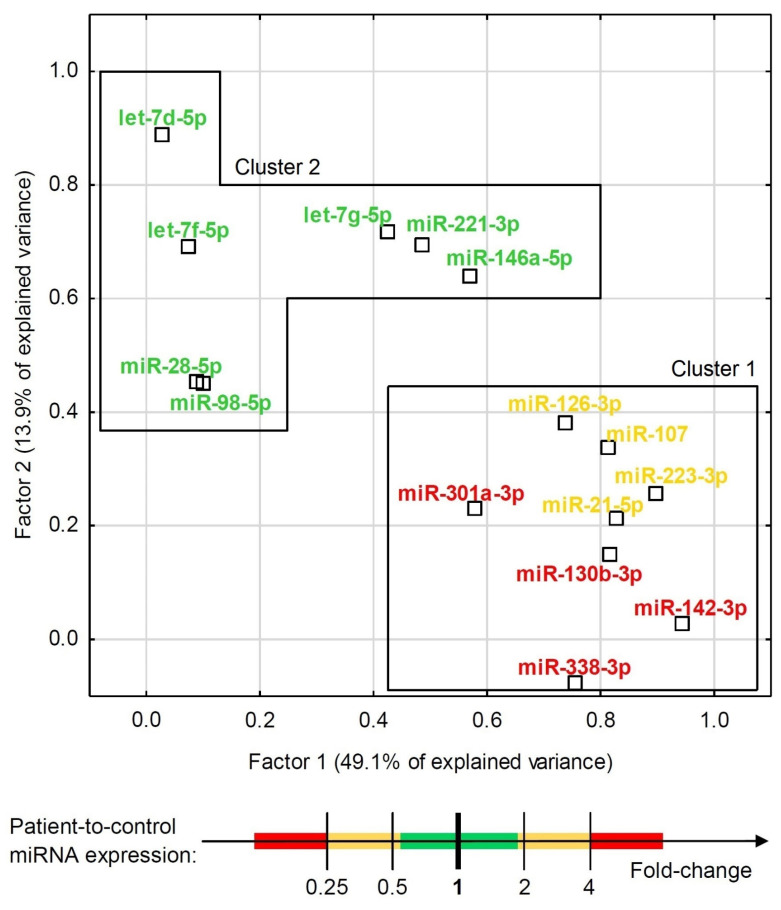
Exploratory factor analysis with factor loadings for expression of all the tested miRNAs in the combined group of ACS patients and healthy controls. The factor differentiation was achieved via raw varimax factor rotation. Two factors accounted for 63.0% of the total variance. MiRNAs are indicated with colors that represent their relative ACS patient-to-control expression.

Although some miRNAs were able to significantly differentiate ACS patients from healthy controls (Figure 2 and Figure 3), the development of a multivariate model for that purpose with the use of miRNAs only was not possible due to their high colinearity. Two orthogonal factors yielded by EFA were able to explain as much as 63.0% of the total variance and all the miRNAs that well-differentiated patients from controls (miR-301a-3p, miR-142-3p, miR-338-3p, miR-130b-3p, miR-107, miR-21-5p, miR-223-3p, and miR-126-3p) substantially loaded the main factor (Figure 4). On the other hand, miRNAs weakly differentiating patients from controls loaded the second factor, with some of them (miR-98-5p, let-7f-5p, let-7d-5p, miR-28-5p) negligibly loading the main one (Figure 4). The Pearson’s *r* correlation matrix with all the tested miRNAs is presented in Supplementary Figure S2, “The correlation matrix with all the tested miRNAs”.

**Figure 5:**
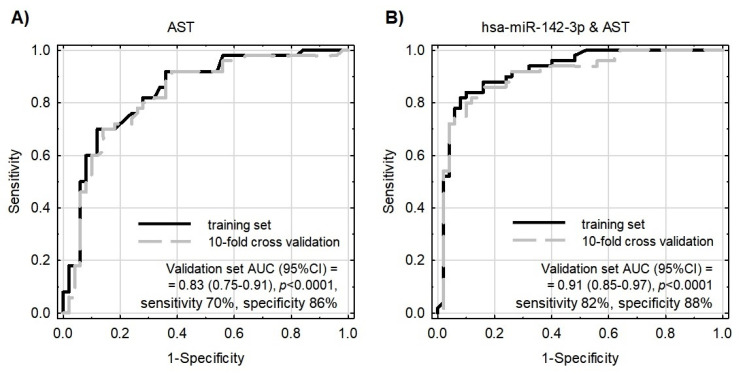
Receiver operating characteristic (ROC) curves for models differentiating ACS patients from healthy controls. Model internal validation was performed with a 10-fold cross-validation technique. (**A**) ROC for the model based on AST only. (**B**) ROC for the model based on miR-142-3p together with AST.

To test whether the evaluated miRNAs could contribute to the better differentiation of patients from controls performed using classical biochemical parameters, the binary logistic regression model was constructed with the use of a step-wise procedure. MiR-142-3p, together with AST, were selected for this model as their correlation was negligible (Pearson’s *r* = 0.067) and both significantly differentiated patients from controls. According to the information criteria and analysis of the coefficients of determination, the model enriched with miR-142-3p performed substantially better in comparison to the model based on AST only (Figure 5 and Table 2). The multivariate model was parsimonious enough not to be prone to overfitting, as evaluated by the validation sample. The model exhibited acceptable goodness-of-fit; however, as Hosmer–Lemeshow statistics presented with borderline *p*-values, this requires further testing and development.

### 3.4. Bioinformatic Analysis of Potential mRNA Targets and Protein–Protein Interactions

To identify the biological significance of tested miRNAs, the bioinformatic analysis focused on the comparison of gene ontology, searching for predictive mRNA targets, and the evaluation of protein–protein interactions (PPIs) was carried out. In the first stage, target genes for statistically significant miRNA molecules were found in GeneSpring Software. To obtain the most reproducible results, the predictive targets of which the presence was confirmed in all studied databases were selected. Furthermore, based on the DAVID Bioinformatic Resources 6.8 [14] and KEGG Pathways [16], signaling pathways and gene ontology for mRNA-miRNA targets were evaluated. To avoid an overabundance of information unrelated to the manuscript topic, only pathways and genes associated with the physiology of platelets were selected. All mRNA targets for particular miRNAs are included in Table 3.

**Figure 6:**
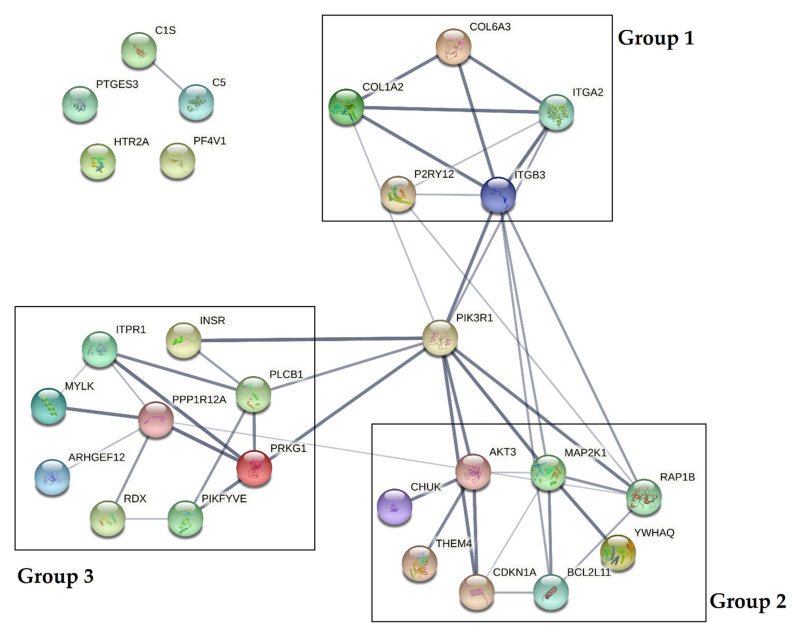
Protein–protein interaction (PPI) analysis of selected proteins present in the platelet proteome. PPIs were determined using the STRING database [18]. The PPI network is presented as lines between nodes, the thickness of which indicates the strength of the interaction.

The obtained results showed that all statistically significant miRNAs may interact with 43 mRNA transcripts present in blood platelets. To test whether crucial interactions between proteins associated with platelet functions are present, protein–protein interaction (PPI) analysis was performed. Of 43 predicted mRNA transcripts, we selected 28 proteins, which were experimentally confirmed in the platelet proteome. A visualization of the PPI network is presented in Figure 6. 

Data obtained from the analysis demonstrated that PPIs could be divided into three separate groups, with a central role of phosphatidylinositol 3-kinase regulatory subunit alpha (PIK3R1). Based on the KEGG Pathways, proteins included in group 1 (COL6A3, COL1A2, ITGA2, ITGB3, P2RY12) were shown to influence the extracellular matrix receptor interaction pathways crucial for platelet adhesion, activation, and aggregation. Proteins included in group 2 (AKT3, MAP2K1, RAP1B, YWHAQ, BCL2L11, CDKN1A, THEM4, CHUK) were shown to affect the PI3K-Akt signaling pathway, and proteins in group 3 (ITPR1, INSR, PLCB1, PRKG1, PIKFYVE, RDX, ARHGEF12, MYLK, PPP1R12A) were shown to be associated with vascular smooth muscle contraction, the calcium signaling pathway during platelet activation, and the regulation of the actin cytoskeleton. Furthermore, three proteins (PTGS3, HTR2A, PF4V1) were not shown to be associated with the studied interactome [16].

The final step of the bioinformatic analysis was focused on the comparison of biological processes and cellular components of predictive mRNA targets of miRNAs included in cluster 1 via Gene Ontology analysis (Supplementary Figures S3 and S4). Data obtained from the DAVID Database [14] revealed significant differences in the mRNA targets of miRNAs in clusters 1 and 2, indicating its multiple roles in the regulation of physiological processes associated with the activation of blood platelets. To identify the most significant potential mRNA targets for miRNAs included in cluster 1 and cluster 2, we decided to verify whether the predictive mRNA targets between both clusters coincided. Furthermore, we tested whether there were differences in the linked targets between clusters and whether particular transcripts were specific to any of the clusters. Based on the obtained results, we selected *ITPR1, PLCB1,* and *PIK3R1* as potential targets of significant miRNAs, that could be used to differentiate between the studied groups.

## 4. Discussion

Our study showed that blood platelets from ACS patients had significantly upregulated expression of eight validated miRNAs: miR-142-3p; miR-107; miR-338-3p, miR-223-3p, miR-21-5p, miR-130b-3p, miR-301a-3p, and miR-221-3p. The presence of all analyzed miRNAs in blood platelets was confirmed in several studies, emphasizing their platelet origin [12,19,20,21]. Due to the complex and multifactorial character of ACS, it is necessary to better understand the molecular mechanisms of increased platelet activity in various areas of disease pathogenesis. The analysis of the platelet miRNA profile in our study was performed with patients presenting the least possible influence of typical risk factors, allowing for the identification and selection of altered expressed miRNA molecules, which could be potentially used to determine the human genetic predisposition to ACS. Furthermore, based on the available literature, we discussed the potential mechanisms of action of eight significant miRNAs to better understand the genetic basis of thrombus formation in blood vessels.

MiR-223-3p was shown to be one of the most abundant miRNAs in blood platelets with a relatively high –ΔCt value. Landry et al. demonstrated that miR-223 was associated with the Ago2 protein and formed a complex with the potential capability to regulate the expression of the P2Y12 receptor, one of the main targets for the pharmacological treatment of patients with thrombotic events. The presence of *P2RY12* mRNA linked with Ago2-miR-223 in blood platelets may suggest the potential influence for de novo protein synthesis, thus influencing the organization of platelet surface receptors through modification of their concentration [12]. Several studies showed that the level of miR-223 was associated with the response of antiplatelet therapy administrated to patients, thus emphasizing its substantial role in the regulation of platelet reactivity [22,23]. Data presented in the mentioned studies showed the opposite results to ours, demonstrating a significantly downregulated level of miR-223 in patients with CVDs. Similarly, there are also other studies presenting the overexpression of miR-223 in CVDs [22,23,24]. The variability of results in studies may arise from the close linkage between the expression of miR-223 and the administration of pharmacotherapy, which influences its level. In our study, blood samples were collected immediately after necessary medical examinations, to avoid potential interactions of medicaments. The overexpression of miR-223 may be thus associated with the short exposition of antiplatelet agents, which could not fully affect the phenotypic changes of platelets. A possible explanation of the increased expression of platelet miR-223 may also be associated with an adaptive reaction to the ischemic event that increases the miR-223 level to inhibit the excessive activation of platelets and P2Y12 receptors.

The disturbed thrombopoiesis and altered functioning of platelets may be triggered by the incorrect regulation of the differentiation of hematopoietic cells. In a study conducted by Chapnik et al., a model of miR-142-knockout mice showed an impaired maturation of megakaryocytes and proplatelet formation, which resulted in thrombocytopenia. Despite disturbed platelet biogenesis, nullified miR-142 mice demonstrated a markedly immature organization of megakaryocytes’ actin and tubulin filaments, the main component of megakaryocytes’ and platelets’ cytoskeleton [24]. Reorganization of the platelet cytoskeleton and the rotation of actin filaments may trigger the release of molecules consisting mostly of an inactive form, i.e., integrin α2bβ3, which is the main receptor for platelet aggregation. Excessive exposition of fibrinogen and vWF binding sites in the mentioned receptor may induce pathogenic thrombosis [25]. Our study showed that of the selected miRNAs, miR-142-3p is one of the most overexpressed in ACS patients compared to healthy controls (FC = 5.16 (95%CI: 2.59–10.28); *p* < 0.0001), both in microarray and RT-qPCR analysis. The role of miR-142-3p overexpression remains unclear; however, the possible mechanism may be analogous to that described by Chapnik et al. [25], indicating that an augmented level of miR-142-3p relates to the disturbed organization of the platelet cytoskeleton, and may cause the uncontrollable formation of actin filaments, including lamelli- and filopodia, which are responsible for recruiting circulating cells during adhesion. Furthermore, stimulated blood platelets may potentially increase the concentration and release of has-miR-142-3p to induce interaction with endothelial cells with the simultaneous stimulation of apoptosis [26].

Moreover, blood platelets play an important role in the pathogenesis of CVDs by disturbing the circadian rhythm. This role may be associated with the genetic background of cardiovascular complications through the diurnal variation in the frequency of ischemic events [27]. Based on bioinformatic analysis, Nagalla et al. showed that miR-107 targets *CLOCK* gene transcripts, thus significantly reducing its expression [20]. Furthermore, Ohkura et al. demonstrated in a mouse model that circadian fluctuation is associated with hemostatic activity. Disturbed circadian rhythm resulted in continuously augmented hemostatic activity in mice. Interestingly, *CLOCK*-mutant mice presented a significantly diminished number of platelets with simultaneous hyperaggregability [28]. The results of our study showed that the expression of platelet miR-107 in ACS patients was nearly three-fold higher compared to the control group (*p* < 0.0001), suggesting that overexpression of miR-107 in blood platelets may be linked with impaired circadian rhythm, and may influence the molecular background of the hyperactivity of blood platelets in CVDs, including ACS.

Highly expressed miRNAs in platelets also include miR-221-3p [29], as was demonstrated in our study. A recent study showed that miR-221-3p is involved in the pathogenesis of primary immune thrombocytopenia (ITP) and is differentially expressed before and after treatment with thrombopoietin receptor agonists (TPO-RAs), indicating its value as a predictive marker of the platelet response to treatment with TPO-RA [30]. Our results showed significant overexpression of miR-221-3p in the blood platelets of ACS patients. Similarly, Ward et al. have reported the significant upregulation of platelet miR-221-3p in patients with ST-segment elevation (STEMI) compared to non-ST-segment elevation (NSTEMI) myocardial infarction. Moreover, miR-221-3p was uniquely associated with platelets and leukocytes in patients with STEMI; however, in NSTEMI patients, miR-221-3p has been revealed to be downregulated in platelets and upregulated in plasma subcomponents [29]. Furthermore, miR-221-3p has been reported as one of the most upregulated circulating miRNAs in patients with acute myocardial infarction (AMI), with a fold change of four and high potential as a prominent predictive biomarker for AMI with AUC = 0.881 (95% CI: 0.774–0.987; *p* = 0.002) [31]. The possible mechanism of action of miR-221-3p in regulating platelet function remains unclear; however, its positive correlation with cardiac troponin in a study by Coskunpinar et al. suggests its high potential of being a biomarker of ACS [31].

Furthermore, miR-21-5p has recently been proposed as a valuable platelet-associated candidate for a novel diagnostic and prognostic biomarker of CVDs, including stroke and pulmonary embolism [32]. Microarray analysis demonstrated that miR-21-5p is one of the most expressed miRNAs in human platelets, cardiac fibroblasts (CFs), endothelial cells (ECs), vascular smooth muscle cells (VSMCs), and cardiomyocytes (CMCs) [32]. Upregulation of this miRNA has been associated with atherosclerosis; thus, its possible application as a diagnostic biomarker for AMI, as well as stroke and pulmonary embolism, has been suggested. MiR-21-5p demonstrated a comparable tendency to that of plasma cardiac troponin I (cTnC) during the early phase of myocardial infarction with a peak at 4 h after the onset of symptoms. Furthermore, plasma miR-21 levels exhibited a significant correlation with cardiac troponin and creatine kinase, which serve as current clinically established markers [33,34]. Moreover, Cenzis et al. reported a significant upregulation of plasma miR-21 in the hypertension cohort compared to healthy controls and an association with systolic and diastolic blood pressure [35]. The direct role of miR-21-5p in platelet function remains unclear; however, based on the KEGG Pathways [16], mRNA targets of miR-21-5p are strongly associated with platelet activation, the PI3K-Akt signaling pathway, focal adhesion, and the regulation of the actin cytoskeleton by affecting e.g., *AKT3, RAP1B, RASGRP1, ITGA2, ITGB3, PIK3R1, PLCB1, THBS2, PDGFRA* genes. Furthermore, the OMIM Database showed an association between miR-21-5p targets and susceptibility to myocardial infarction [15].

In the case of miR-301a-3p, miR-130b-3p, and miR-338-3p, there is a lack of reported information concerning the possible mechanism of action in blood platelets and their role in thrombosis. Our microarray analysis showed that miR-301a-3p, miR-130b-3p, and miR-338-3p were substantially upregulated in ACS patients. RTq-PCR confirmed these results, demonstrating 5.52-fold, 4.11-fold, and 4.31-fold augmented expression within ACS patients, respectively. To assess their molecular function that could influence thrombosis, we found possible mRNA targets using GeneSpring software, and based on the Gene Ontology Database in DAVID [14] we compared the biological processes linked with targeted mRNA. Our analysis showed that transcripts targeted by miR-301a-3p, miR-130b-3p, and miR-338-3p in the case of thrombosis are responsible, e.g., for the signal transduction process, the regulation of GTPase activity, platelet activation, phosphatidylinositol phosphorylation, response to hypoxia, and wound healing. However, to confirm their association with these mentioned processes, further detailed analysis is required.

The influence of miRNAs on the post-transcriptional regulation of gene expression may have a great impact on altering physiological processes to pathological conditions. To assess the biological significance of those alterations, mRNA targets for statistically significant miRNAs were found, and the potential PPI network analysis of transcripts encoded by these genes was performed (Table 3, Figure 6). Our results showed that all analyzed proteins could be clustered in three separate groups, associated with several signaling pathways. Group 1 consisted of COL6A3, COL1A2, ITGA2, ITGB3, and P2RY12, which may influence the extracellular matrix receptor interaction pathways crucial for platelet adhesion and activation. Collagens (COL6A3 and COL1A2) are key activators of glycoprotein (GP) VI, and GP Ia/IIa (ITGA2 is an alias for GP Ia), and are considered to be prothrombotic factors [36]. Their presence at the mRNA and protein level was confirmed in a study by Huang et al. [17]. Collagens I–IV support the adhesion of platelets in high shear forces, thus inducing platelet aggregation in small arteries; however, collagens type VI–VIII are less reactive due to the lower shear rates [37]. The presence of collagens and their receptors is crucial for the proper adhesion of platelets to the endothelium. ITGB3 (GP IIIa) is one of the two subunits that form the most crucial platelet integrin, GP IIb/IIIa, which plays a key role in the aggregation process. Signal transduction in the activation of blood platelets from various receptors leads to changes in the conformation of GP IIb/IIIa, which augment its affinity for fibrinogen [16,38]. P2RY12 is one of the most important platelet surface receptors and constitutes the main target of antiplatelet therapy [2]. Activation by adenosine diphosphate (ADP) causes the inhibition of adenylyl cyclase, which decreases the level of cyclic adenosine monophosphate (cAMP) and leads to platelet aggregation. P2RY12 also influences the PI3K-Akt signaling pathway, thus providing the conformational changes of GP IIb/IIIa [39]. Disturbances in the expression of molecules associated with the extracellular matrix interaction pathways may result in the alteration of platelet activity, thus enhancing their impact on thrombosis. Proteins included in group 2 (AKT3, MAP2K1, RAP1B, YWHAQ, BCL2L11, CDKN1A, THEM4, and CHUK) present a strong association with the PI3K-Akt signaling pathway. Phosphatidylinositol 3-kinases (PI3Ks) are enzymes that phosphorylate phosphatidylinositol 4,5-biphosphate (PIP2) to phosphatidylinositol 3,4,5-triphosphate (PIP3). Furthermore, PIP3 links with protein kinase B (Akt) which induces signal transduction to platelet activation via von Willebrand factor receptor GP Ib-IX-V, collagen-associated GP VI, and GPCRs (i.e., P2Y12). Signal transduction occurs downstream by Rap1b to interact with talin and kindlin, two proteins responsible for the conformational changes of GP IIb/IIIa [40]. The occurrence of disorders affecting any of the factors involved in the complex PI3K-Akt signaling pathway may lead to platelet dysfunction during physiological hemostasis and/or pathological thrombosis. Group 3, consisting of ITPR1, INSR, PLCB1, PRKG1, PIKFYVE, RDX, ARHGEF12, MYLK, and PPP1R12A proteins, was shown to interact with the vascular smooth muscle contraction calcium signaling pathway during platelet activation, and to participate in regulation of actin cytoskeleton. Changes in the calcium levels during platelet activation may lead to the activation of actin–myosin interaction, Rap1 and PI3K-Akt pathway signal transduction, thromboxane A2 (THX2) synthesis, granule secretion, and phospholipase (PL) activity, ensuring the occurrence of processes crucial for platelet functions [41]. VSMCs are the most abundant type of arterial wall cells. Through vasodilatation and vasoconstriction, VSMCs maintain vascular homeostasis and physiological interactions with morphotic elements. Dysregulation in the functioning of VSMCs may lead to the pathogenesis of various cardiovascular diseases, which may directly or indirectly influence ACS [42]. Furthermore, the disturbed regulation of the actin cytoskeleton in blood platelets may affect the process of shape change and the secretion of biologically active molecules stored in granules [42]. All proteins included in the three groups may directly or indirectly interact with PI3KR1, potentially affecting all crucial pathways in platelet activation. The confirmation of our bioinformatics analysis requires further detailed studies, focused not only on the miRNA levels but also on other molecular factors with the ability to regulate post-transcriptional gene expression.

One of the major findings in our study was accomplished through EFA analysis, which demonstrated that the studied miRNAs could be classified into two unrelated clusters: cluster 1, represented by miRNAs significantly differentiating ACS patients from healthy donors, and cluster 2, which consisted of miRNAs which did not contribute to the differentiation of ACS patients from controls, among others. Furthermore, the EFA analysis suggested that there is no interplay between the expression of miRNAs of both cluster groups and the groups may provide independent BFs (Figure 2). To determine these differences, we performed a comparative Gene Ontology analysis of mRNA transcripts controlled by the analyzed miRNAs, with the determination of their BFs. Thus, we identified and summed up the number of genes responsible for performing a specific BF and compared the number of genes between both groups using the DAVID Database [14]. Our results showed that mRNAs targeted by miRNAs included in cluster 1, capable of differentiating ACS patients from healthy donors, presented a seven-fold higher number of transcripts for genes associated with the positive regulation of GTPase activity and positive regulation of PLC activity, over a six-fold elevated number of genes linked with the response to hypoxia, a nearly five-fold higher number of genes related to the regulation of chemotaxis and signaling of phosphatidylinositol 3-kinase, and a more than two-times increased number of genes associated with signal transduction, response to drugs, and the regulation of nitric oxide biosynthesis (Supplementary Figure S3, “Heatmap with Gene Ontology: biological function analysis”. Moreover, we studied cellular components (CCs) in the Gene Ontology overview to identify the potential location relative to cellular structures in which a particular gene performs its function. CC analysis showed that in cluster 1, a strikingly higher number of genes is associated with cytosol, the plasma membrane, integrin complex, and cytoskeleton; however, in cluster 2, a significantly elevated number of genes was associated with the endoplasmic reticulum lumen, collagen trimers, proteinaceous extracellular matrix, filopodia, and lamellipodia (Supplementary Figure S4, “Heatmap with Gene Ontology: cellular compartment analysis”). Our analysis demonstrated that the miRNAs that are able to differentiate ACS patients from healthy donors, included in cluster 1, regulate genes associated with the physiology of blood platelets, including their activation in various signaling pathways, shape change, response to pathological conditions, and medications, and overall signal transduction. However, miRNAs in cluster 2 that do not contribute to distinguishing between the study and control group may be associated with the interaction of platelets in the vascular environment without significantly interfering with platelets’ physiological processes.

Using the DAVID database, we assigned genes regulated by the analyzed miRNAs to those BFs for which the difference in the number of genes was from two-fold to seven-fold (Supplementary Figure S3). Furthermore, genes belonging to the predictive transcriptome-proteome profile of blood platelets were selected [17]. Subsequent comparative analysis of the number of selected genes associated with particular BFs allowed the selection of mRNA transcripts showing the greatest differences in frequency between cluster 1 and cluster 2. We decided to select *ITPR1*, *PLCB1*, and *PIK3R1* transcripts as targets for miRNAs in Cluster 1.

In the platelet activation pathway, a variety of factors are required for proper cellular responses, including adhesion, degranulation, and the expression of coagulant activity [43]. One of the central steps in these processes is the elevation of calcium ions (Ca^2+^) which may be initiated by soluble agonists such as thrombin, ADP, and TXA2. The further stimulation of receptors that bind to G proteins of GPCRs leads to the activation of PLCβ, encoded by *PLCB1* [43]. In platelets, PLCβ, together with PLCγ2, releases inositol 1,4,5-triphosphate (IP3), which mediates calcium mobilization, acting on the ion channel of inositol 1,4,5-triphosphate receptor type 1 (IP3R1), encoded by the *ITPR1* gene [44,45]. IP3 is generated through PLC-mediated hydrolysis of PIP2 and initiates the release of calcium ions from the platelet-dense tubular system. PLCβ mediates this process after the stimulation of GPCRs, whereas PLCγ hydrolyzes PIP2 after the stimulation of GP VI by collagen [46]. PI3K enhances the activation of PLCγ and thus increases the hydrolysis of PIP2 mediated by GP VI [46]. Calcium transporters are very sensitive to oxygen deficiency, and hypoxia has been shown to modulate *IP3R1* gene expression differently [47]. The *PIK3R1* gene is essentially important in the context of our bioinformatic analysis, as it encodes a protein of which a critical role in cardiovascular physiology and pathology has been widely proposed [48]. Furthermore, our PPI analysis showed its central role in the studied interactome. Lurong et al. suggested that PI3K contributes to mediating a thrombin-stimulated elevation of the cytosolic calcium level in platelets, mediated through PLCγ [46]. PI3K enzymes support platelet activation and thrombosis, thus serving as promising candidates for the prevention of thrombus formation [48].

Due to the fact that statistically significant overexpressed miRNAs are strongly correlated with each other (EFA analysis and Supplementary Material Figure S2) and gathered in cluster 1, the construction of a multivariate model composed only of miRNAs would be incorrect and fail during testing. For this reason, we decided to verify miR-142-3p, which presents the best sensitivity and specificity in differentiating studied groups (sensitivity: 64%, specificity: 76%, AUC = 0.75 (0.65–0.84); *p* < 0.0001) in combination with non-correlated AST. AST is a member of the aminotransferases, a family of enzymes responsible for maintaining the metabolism of amino acids by transferring amino groups. Studies showed that the AST level starts to increase in post-ACS patients 6–8 h after the ischemic event occurs, and remains increased up to 36 h. Research over the last two decades has showed widely varying results concerning its usage as a biomarker of ACS, with gradually reduced epidemiological interest, especially in the presence of more sensitive biomarkers, including troponins [49]. However, the role of AST as a predictive marker in combination with transcriptomic factors, including miRNAs, was not evaluated until the present report. Our data demonstrated that AST levels were not correlated with miRNA levels and had promising potential in the diagnostic model (AUC = 0.83). However, the addition of miR-142-3p to the AST-based model showed substantially improved quality of patient–control differentiation (AUC = 0.91). The advantage of the combined model (miR-142-3p and AST) is that it is highly accurate in differentiating ACS patients from control donors, which is indicated by pseudo-R2 values (Table 3). Moreover, the combined model better describes our result, which can be proven by the decreased values of information criteria, and a well-fitted validation set, which simultaneously indicate a very important role of platelet miR-142-3p as a potential biomarker of ACS predisposition.

Given the study design, it was impossible to avoid certain study limitations. First, blood platelets contain a very small amount of nucleic acids compared to other blood cells. Despite taking great caution to avoid possible contaminations of samples, we cannot fully exclude the presence of non-platelet-origin miRNAs in the screening analysis. Furthermore, due to the high correlations of selected miRNAs, we could not fully evaluate their potential role as biomarkers for ACS prediction. Further studies including a larger number of miRNAs and a larger population are required to create a functional model that differentiates patients from controls. Moreover, there is currently no standardized, documented validation model for measuring miRNA expression by means of RT-qPCR. Due to the use of different endogenous and/or exogenous controls, results compared between studies may not be fully accurate. We are fully aware that using the term “prognostic biomarker” in our study should also contain results from miRNA profiling in the population of patients before ischemia occurs; however, the genetic alterations associated with disturbed transcriptomic processes demonstrated in our study may indicate a molecular background of augmented platelet reactivity, which may be linked with induced thrombosis, which results in ACS.

## 5. Conclusions

To conclude, the results of our study showed a significantly increased expression of miR-142-3p; miR-107; miR-338-3p, miR-223-3p, miR-21-5p, miR-130b-3p, miR-301a-3p, and miR-221-3p in the group of ACS patients compared to healthy volunteers. Furthermore, the analyzed platelet miRNAs appear to constitute two unrelated clusters, responsible for distinct BFs. Bioinformatic analysis showed that miRNAs in cluster 1 may be associated with the physiological processes of blood platelets, and miRNAs in cluster 2 may be linked with platelet–vascular environment interactions. Our analysis based on the functional annotations and signaling pathways revealed that *ITPR1*, *PLCB1*, and *PIK3R1* may constitute predictive targets of miRNAs in cluster 1. Furthermore, PPI analysis showed that proteins, of which the expression may be regulated by statistically significant miRNAs, form three groups associated with the extracellular matrix receptor interaction pathways crucial for platelet adhesion, activation, and aggregation (group 1); the PI3K-Akt signaling pathway (group 2); and vascular smooth muscle contraction, the calcium signaling pathway during platelet activation, and the regulation of actin cytoskeleton (group 3). Moreover, based on the ROC curves, we showed that miR-142-3p presents a high potential to distinguish ACS patients from healthy donors; however, in combination with AST, the combined model was able to distinguish ACS patients from controls with 82% sensitivity, 88% specificity with AUC = 0.91 (0.85–0.97); *p* < 0.0001. The data obtained in our study show that miR-142-3p has a great potential to be used as a prognostic biomarker of ACS predisposition and/or to improve the quality of existing biomarkers.

## Figures and Tables

**Table 1:** Clinical characteristics of the ACS patients and healthy volunteers.

Clinical Parameter	Study Group	Control Group	References
Age	54 (46–59.25)	50 (40.75–57.5)	-
Male	43	40	-
Female	7	10	-
Erythrocytes (10^6^/µL)	4.57 (4.283–4.988)	4.95 (4.545–5.27)	4.2–6.1
Leukocytes (10^3^/µL)	8.6 (7.333–9.648)	6.165 (4.828–7.935)	4–11
Blood platelets (10^3^/µL)	251.5 (201.8–281.8)	250 (215–299.8)	150–400
Glucose (mmol/l)	6 (5.365–6.535)	5.055 (4.745–5.563)	4.1–5.1
Creatinine (µmol/l)	82.5 (74.5–91)	74.26 (68.51–86.85)	64–104
GFR (ml/min/1.73m^2^)	95.2 (79.13–101)	86.33 (79.93–99.01)	>60
Cholesterol (mmol/l)	5.645 (4.475–6.81)	4.8 (4.075–5.303)	3–5
HDL (mmol/l)	1.145 (1–1.333)	1.350 (1.120–1.810)	>1
LDL (mmol/l)	3.21 (2.373–4.41)	2.780 (2.220–3.238)	-
Triglycerides (mmol/l)	1.805 (1.155–2.815)	1.195 (0.938–1.688)	<1.7
AST (U/I)	32.00 (25.75–43.65)	19.55 (16.30–25.85)	0–50
ALT (U/I)	29 (21–39)	20.85 (14.73–33.35)	0–50
TSH (mIU/l)	1.8 (1.183–2.75)	2.055 (1.413–2.863)	0.27–4.20
BMI	<35	<35	<35

Clinical parameters are presented as a median and 1st–3rd quartile of 25th–75th percentile. Abbreviations: ALT—alanine transaminase; AST—aspartate transaminase; BMI—body mass index. GFR—glomerular filtration rate; HDL—high-density lipoprotein; LDL—low-density lipoprotein; TSH—thyroid-stimulating hormone; UA—unstable angina.

**Table 2:** Characteristics of the binary logistic regression models differentiating ACS patients from healthy controls based on AST only and miR-142-3p together with AST.

Model Characteristics	Model Based on AST	Model Based on miR-142-3p and AST
Odds ratio (95%CI), *p*-value		
AST (Box–Cox transformed*100) ^1^	2.08 (1.54–2.79), *p* < 0.0001	2.57 (1.73–3.81), *p* < 0.0001
miR-142-3p (−ΔCt)	N/A	1.91 (1.37–2.67), *p* = 0.0001
Coefficients of determination:		
Cox–Snell R^2^	32.5%	48.2%
Nagelkerke R^2^	43.4%	64.2%
Information criteria:		
AIC	103.3	78.9
BIC	108.5	86.7
Goodness-of-fit:		
Hosmer–Lemeshow χ^2^(df), *p*-value	6.60 (8), *p* = 0.58	14.84 (8), *p* = 0.062

AST—aspartate transaminase, N/A—not applicable, AIC—Akaike information criterion, BIC—Bayesian information criterion; ^1^ Box–Cox λ = −0.9071.

**Table 3:** Summary of predictive mRNA-miRNA targets associated with the central biological functions of blood platelets.

MicroRNA	mRNA Targets ^1^	Kegg Pathway ^2^	Associated Genes
miR-223-3p	46	PI3K-Akt signaling pathway	AKT3, CREB1 *, CHUK, FGF2 *, ITGA2, PDGFRA *
		Platelet activation	AKT3, RASGRP1 *, ITGA2, P2RY12
miR-142-3p	41	Platelet activation	AKT3, RASGRP1 *, ARHGEF12, COL24A1 *, ITGA2, MYLK, PLCB1, PPP1R12A
		Regulation of actin cytoskeleton	ARHGEF12, FGF2 *, ITGA2, MYLK, PPP1R12A
		Focal adhesion	AKT3, COL24A1 *, ITGA2, MYLK, PPP1R12A
		Vascular smooth muscle contraction	*ARHGEF12, MYLK, PLCB1, PPP1R12A*
miR-21-5p	58	Platelet activation	AKT3, RAP1B, RASGRP1 *, ARHGEF12, COL3A1, COL24A1 *, ITPR1, ITGA2, ITGB3, PIK3R1, PLCB1, PRKG1
		PI3K-Akt signaling pathway	AKT3, PHLPP2 *, COL3A1, COL24A1 *, CHUK, FGF2 *, ITGA2, ITGB3, PIK3R1, PDGFRA *,
miR-107	92	PI3K-Akt signaling pathway	AKT3, BCL2L11 *, PHLPP2 *, CREB1 *, CHRM2 *, COL3A1, COL6A3, COL24A1 *, FGF2 *, INSR, ITGA2, MAP2K1, PIK3R1, VEGFA *
		Platelet activation	AKT3, COL3A1, COL24A1 *, ITPR1, ITGA2, MYLK, PIK3R1, PLCB1, PPP1R12A
		Focal adhesion	AKT3, COL3A1, COL6A3, COL24A1 *, ITGA2, MAP2K1, MYLK, PIK3R1, PPP1R12A, VEGFA *
		cAMP signaling pathway	AKT3, ADRB2 *, CREB1*, CHRM2 *, GRIN2A *, MAP2K1, PIK3R1, PPP1R12A
		Regulation of actin cytoskeleton	CHRM2 *, FGF2 *, ITGA2, MAP2K1, MYLK, PIK3R1, PPP1R12A
		Calcium signaling pathway	HTR2A, ADRB2 *, CHRM2 *, GRIN2A *, ITPR1, MYLK, PLCB1
		Circadian entrainment	CREB1 *, GRIN2A *, ITPR1, PLCB1, PRKG1
		Phosphatidylinositol signaling system	*ITPR1, PIK3R1, PLCB1*
		Chemokine signaling pathway	*AKT3, MAP2K1, PIK3R1, PLCB1, PF4V1*
miR-221-3p	71	PI3K-Akt signaling pathway	AKT3, BCL2L11 *, PHLPP2 *, CREB1 *, CHRM2 *, ITGA2, ITGB3, MAP2K1, PIK3R1, PDGFRA *, THEM4, YWHAQ
		Regulation of actin cytoskeleton	CHRM2 *, ITGA2, ITGB3, MAP2K1, PIKFYVE, PIK3R1, PDGFRA *, RDX
		Platelet activation	AKT3, RAP1B, RASGRP1 *, ITGA2, ITGB3, PIK3R1, PLCB1, PRKG1
		Focal adhesion	AKT3, RAP1B, ITGA2, ITGB3, MAP2K1, PIK3R1, PDGFRA *
		cAMP signaling pathway	AKT3, RAP1B, CREB1 *, CHRM2 *, MAP2K1, PIK3R1
miR-301a-3p	91	PI3K-Akt signaling pathway	AKT3, BCL2L11, PHLPP2 *, CREB1 *, CHRM2 *, COL1A2, COL6A3, CHUK, CDKN1A, INSR, MAP2K1, PIK3R1, PDGFRA *
		Platelet activation	AKT3, ARHGEF12, COL1A2, ITPR1, PIK3R1, PLCB1, PRKG1, PLAU *
		cAMP signaling pathway	AKT3, RAPGEF4 *, ADRB1 *, CREB1 *, CHRM2 *, MAP2K1, PIK3R1
		Focal adhesion	AKT3, COL1A2, COL6A3, MAP2K1, PIK3R1, PDGFRA *
		Regulation of actin skeleton	ARHGEF12, CHRM2 *, MAP2K1, PIKFYVE, PIK3R1, PDGFRA *, RDX
		Vascular smooth muscle contraction	*ARHGEF12, ITPR1, MAP2K1, PLCB1, PRKG1*
		Calcium signaling pathway	ADRB1 *, CHRM2 *, ITPR1, PLCB1, PDGFRA *
		Phosphatidylinositol signaling system	*ITPR1, PIKFYVE, PIK3R1, PLCB1*
		Arachidonic acid metabolism	*PTGES3*
miR-130b-3p	93	PI3K-Akt signaling pathway	AKT3, BCL2L11, PHLPP2 *, CREB1 *, CHRM2 *, COL1A2, COL6A3, CHUK, CDKN1A, INSR, MAP2K1, PDGFRA *
		Platelet activation	*AKT3, RAP1B, ARHGEF12, COL1A2, ITPR1, MYLK, PLCB1, PRKG1*
		Regulation of actin cytoskeleton	ARHGEF12, CHRM2 *, MAP2K1, MYLK, PIKFYVE, PDGFRA *, RDX
		cAMP signaling pathway	AKT3, RAP1B, RAPGEF4 *, ADRB1 *, CREB1 *, CHRM2 *, MAP2K1
		Vascular smooth muscle contraction	*ARHGEF12M ITPR1, MAP2K1, MYLK, PLCB1, PRKG1*
		Complement and coagulation cascade	F3 *, C1S, C5, PLAU*
		Arachidonic acid metabolism	*PTGES3*
miR-338-3p	58	PI3K-Akt signaling pathway	AKT3, PHLPP2 *, CREB1 *, CHRM2 *, COL6A3, FGF2 *, ITGB3, PDGFRA *
		Regulation of actin cytoskeleton	ARHGEF12, CHRM2 *, FGF2 *, ITGB3, PDGFRA *, RDX
		Platelet activation	*AKT3, ARHGEF12, ITPR1, ITGB3, PRKG1*
		Calcium signaling pathway	ADRB2 *, CHRM2 *, GRIN2A *, ITPR1, PDGFRA *
		cAMP signaling pathway	AKT3, ADRB2 *, CREB1 *, CHRM2 *, GRIN2A *
		Focal adhesion	AKT3, COL6A3, ITGB3, PDGFRA *

^1^^,2^ Numbers presented in rows show the overall number of mRNA targets found in the 6 databases: miRDB, microRNAorg, PITA, PICTAR, TARBASE, and TARGETSCAN in GeneSpring software. * The presence of mRNA transcripts was confirmed in blood platelets, without protein products [17]. Underlined—The presence of protein was confirmed in blood platelets, without mRNA transcripts [17].

## Data Availability

All data obtained from this study are included in the manuscript.

## Supplementary Materials

The following are available online at https://www.mdpi.com/article/10.3390/cells10123526/s1, Supplementary Figure S1: The heatmap of miRNAs expression in microarray analysis; Supplementary Figure S2: The correlation matrix with all the tested miRNAs; Supplementary Figure S3: Heatmap with Gene Ontology: biological function analysis; Supplementary Figure S4: Heatmap with Gene Ontology: cellular compartment analysis.
