# Supplementary material for: Screening Analysis of Platelet miRNA Profile Revealed miR-142-3p as a Potential Biomarker in Modeling the Risk of Acute Coronary Syndrome
Source: Cells. 2021 Dec 14;10(12):3526. doi: 10.3390/cells10123526 (PMC8700136; doi:10.3390/cells10123526)
Supplement: Supplementary file 1 [file cells-10-03526-s001.zip › Supplementary Figure S2.pdf]

Supplementary Figure S2. The correlation matrix with all the tested miRNAs

Pearson's r	hsa-miR-301a-3p	hsa-miR-142-3p	hsa-miR-338-3p	hsa-miR-130b-3p	hsa-miR-107	hsa-miR-21-5p	hsa-miR-223-3p	hsa-miR-126-3p	hsa-miR-98-5p	hsa-miR-221-3p	hsa-miR-146a-5p	hsa-let-7f-5p	hsa-let-7d-5p	hsa-miR-28-5p	hsa-let-7g-5p	
hsa-miR-301a-3p		0,475	0,402	0,484	0,545	0,451	0,512	0,442	0,169	0,379	0,430	0,186	0,281	0,119	0,364	MiRNAs with fold-change >4
hsa-miR-142-3p	0,475		0,668	0,728	0,808	0,793	0,854	0,676	0,164	0,450	0,567	0,072	0,068	0,091	0,425	
hsa-miR-338-3p	0,402	0,668		0,603	0,570	0,498	0,615	0,453	0,082	0,243	0,278	0,269	-0,009	0,014	0,255	
hsa-miR-130b-3p	0,484	0,728	0,603		0,704	0,604	0,749	0,603	0,245	0,441	0,514	0,135	0,208	0,207	0,419	
hsa-miR-107	0,545	0,808	0,570	0,704		0,639	0,806	0,677	0,311	0,582	0,664	0,229	0,391	0,268	0,500	MiRNAs with fold-change of 1.8-4
hsa-miR-21-5p	0,451	0,793	0,498	0,604	0,639		0,801	0,651	0,159	0,624	0,647	0,223	0,075	0,162	0,588	
hsa-miR-223-3p	0,512	0,854	0,615	0,749	0,806	0,801		0,780	0,257	0,602	0,658	0,250	0,258	0,214	0,511	
hsa-miR-126-3p	0,442	0,676	0,453	0,603	0,677	0,651	0,780		0,134	0,624	0,681	0,258	0,354	0,249	0,589	
hsa-miR-98-5p	0,169	0,164	0,082	0,245	0,311	0,159	0,257	0,134		0,116	0,144	0,362	0,383	0,145	0,300	MiRNAs with fold-change <1.8
hsa-miR-221-3p	0,379	0,450	0,243	0,441	0,582	0,624	0,602	0,624	0,116		0,750	0,439	0,568	0,315	0,769	
hsa-miR-146a-5p	0,430	0,567	0,278	0,514	0,664	0,647	0,658	0,681	0,144	0,750		0,385	0,601	0,343	0,645	
hsa-let-7f-5p	0,186	0,072	0,269	0,135	0,229	0,223	0,250	0,258	0,362	0,439	0,385		0,499	0,138	0,528	
hsa-let-7d-5p	0,281	0,068	-0,009	0,208	0,391	0,075	0,258	0,354	0,383	0,568	0,601	0,499		0,359	0,573	
hsa-miR-28-5p	0,119	0,091	0,014	0,207	0,268	0,162	0,214	0,249	0,145	0,315	0,343	0,138	0,359		0,153	
hsa-let-7g-5p	0,364	0,425	0,255	0,419	0,500	0,588	0,511	0,589	0,300	0,769	0,645	0,528	0,573	0,153		
	MiRNAs with fold-change >4				MiRNAs with fold-change of 1.8-4				MiRNAs with fold-change <1.8							

The matrix presents the values of Pearson's r correlation coefficients for all pairs of expressions of the tested miRNAs. The matrix cells are colored according to the Pearson's r values. The most top-left set of four miRNAs depicts these with extreme expression fold-change (higher than 4 – corresponding to red-colored miRNAs in Figure 4). The next top-left set of four miRNAs depicts these with intermediate expression fold-change (1.8-4 – corresponding to yellow-colored miRNAs in Figure 4). The rest of miRNAs presented in the bottom and right part of the matrix depicts these with expression fold-change lower than 1.8 (corresponding to green-colored miRNAs in Figure 4). The Pearson's r matrix informs that miRNAs that substantially differentiate ACS patients from healthy controls are generally more correlated with each other than miRNAs not differing between ACS patients and controls. This corresponds to the exploratory factor analysis presented in Figure 4.
