# Supplementary material for: Screening Analysis of Platelet miRNA Profile Revealed miR-142-3p as a Potential Biomarker in Modeling the Risk of Acute Coronary Syndrome
Source: Cells. 2021 Dec 14;10(12):3526. doi: 10.3390/cells10123526 (PMC8700136; doi:10.3390/cells10123526)
Supplement: Supplementary file 1 [file cells-10-03526-s001.zip › Supplementary Figure S3 and S4.pdf]

Supplementary Figure S3. Heatmap with Gene Ontology: Biological Functions analysis. Numbers in rows presents number of genes associated with particular function.

**Biological Function**	**Cluster 1**	**Cluster 2**
signal transduction	102	47
positive regulation of transcription from RNA polymerase II promoter	67	36
positive regulation of GTPase activity	53	7
positive regulation of cell migration	40	33
response to drug	35	17
extracellular matrix organization	33	40
aging	29	31
protein phosphorylation	28	42
negative regulation of gene expression	24	0
phosphatidylinositol phosphorylation	23	6
positive regulation of smooth muscle cell proliferation	22	5
positive regulation of ERK1 and ERK2 cascade	22	4
MAPK cascade	22	4
response to hypoxia	19	3
activation of MAPK activity	19	0
cerebral cortex development	18	17
regulation of phosphatidylinositol 3-kinase signaling	18	4
platelet activation	16	15
collagen catabolic process	15	26
phosphatidylinositol-mediated signaling	15	3
positive regulation of phospholipase C activity	15	2
peptidyl-tyrosine phosphorylation	14	3
positive regulation of positive chemotaxis	14	3
positive regulation of nitric oxide biosynthetic process	13	6
cell adhesion	13	0
protein stabilization	12	6
chemotaxis	12	0
response to cold	11	18
positive regulation of MAP kinase activity	11	9

positive regulation of cell proliferation	11	5
cell adhesion mediated by integrin	11	2
cellular response to estradiol stimulus	10	2
nervous system development	10	0
positive regulation of gene expression	10	0
positive regulation of protein complex assembly	9	2
leukocyte chemotaxis	9	0
phosphatidylinositol-3-phosphate biosynthetic process	9	0
positive regulation of protein kinase B signaling	9	0
cell-matrix adhesion	9	0
temperature homeostasis	8	12
cellular response to insulin-like growth factor stimulus	8	2
sphingolipid biosynthetic process	8	0
insulin-like growth factor receptor signaling pathway	8	0
G-protein coupled acetylcholine receptor signaling pathway	8	0
positive regulation of protein phosphorylation	7	12
positive regulation of angiogenesis	7	3
positive regulation of developmental growth	6	6
release of sequestered calcium ion into cytosol	6	3
positive regulation of peptidyl-tyrosine phosphorylation	6	3
positive regulation of cell adhesion	6	2
response to muscle activity	6	2
negative regulation of protein phosphorylation	6	0
integrin-mediated signaling pathway	6	0
positive regulation of leukocyte migration	6	0
cellular glucose homeostasis	6	0
skeletal system development	4	8
response to superoxide	4	8
fatty acid oxidation	4	2
regulation of immunoglobulin secretion	4	0
glutamate receptor signaling pathway	4	0
regulation of smooth muscle contraction	4	0

cellular response to amino acid stimulus	3	12
positive regulation of cysteine-type endopeptidase activity involved in apoptotic process	3	0
positive regulation of endothelial cell migration	3	0
positive regulation of cell division	3	0
aorta smooth muscle tissue morphogenesis	2	10
positive regulation of glycolytic process	2	4
smooth muscle contraction	2	4
positive regulation of lipophagy	2	0
inositol phosphate biosynthetic process	2	0
regulation of phosphatidylinositol 3-kinase activity	2	0
regulation of establishment of endothelial barrier	2	0
positive regulation of protein kinase C signaling	2	0
tube formation	2	0
phosphatidylinositol metabolic process	2	0
sleep	2	0
cell migration involved in sprouting angiogenesis	2	0
positive regulation of blood vessel endothelial cell migration	2	0
positive regulation of focal adhesion assembly	2	0

Supplementary Figure S4. Heatmap with Gene Ontology: Cellular Compartment analysis. Numbers in rows presents number of genes associated with particular function.

**Cellular Compartment**	**Cluster 1**	**Cluster 2**
cytoplasm	72	46
membrane	71	13
extracellular region	58	68
extracellular space	51	43
cytosol	48	0
plasma membrane	43	16
Golgi apparatus	42	34
proteinaceous extracellular matrix	23	36
collagen trimer	21	38
extracellular matrix	20	14
external side of plasma membrane	20	0
receptor complex	16	27
cytoskeleton	15	4
intracellular	15	0
membrane raft	14	3
nuclear euchromatin	12	2
endoplasmic reticulum lumen	11	40
integral component of plasma membrane	10	9
extrinsic component of membrane	9	0
early endosome	8	16
endoplasmic reticulum	8	0
focal adhesion	5	0
lamellipodium	4	8
integrin complex	4	0
filopodium	3	6
cell-cell junction	3	3
intrinsic component of plasma membrane	2	2
CD40 receptor complex	2	0
collagen type IV trimer	0	8
organelle membrane	0	6
collagen type VI trimer	0	4
endosome membrane	0	3
