# Supplementary figures and images for: Screening Analysis of Platelet miRNA Profile Revealed miR-142-3p as a Potential Biomarker in Modeling the Risk of Acute Coronary Syndrome
Source: Cells. 2021 Dec 14;10(12):3526. doi: 10.3390/cells10123526 (PMC8700136; doi:10.3390/cells10123526)

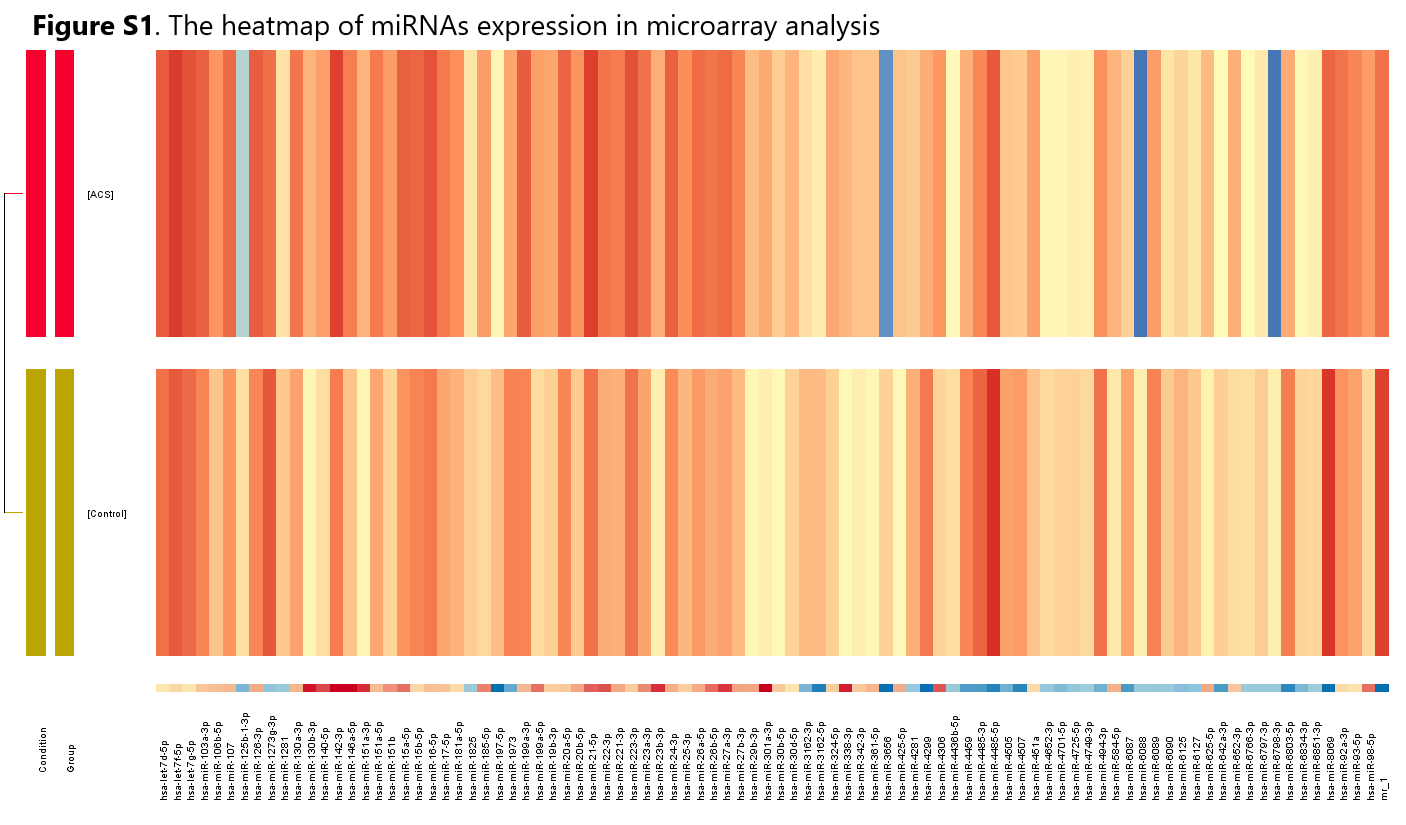

Supplement: Supplementary file 1 [file cells-10-03526-s001.zip › Supplementary Figure S1.tiff]
